# Inhibition of the mRNA-Binding Protein IGF2BP1 Suppresses Proliferation and Sensitizes Neuroblastoma Cells to Chemotherapeutic Agents

**DOI:** 10.3389/fonc.2021.608816

**Published:** 2021-03-16

**Authors:** Jason M. Biegel, Mayura Dhamdhere, Shuang Gao, Chethana P. Gowda, Yuka Imamura Kawasawa, Vladimir S. Spiegelman

**Affiliations:** ^1^ Division of Hematology and Oncology, Pediatric Department, Penn State College of Medicine, Hershey, PA, United States; ^2^ Department of Public Health Sciences, Penn State College of Medicine, Hershey, PA, United States; ^3^ Departments of Pharmacology and Biochemistry and Molecular Biology, Penn State College of Medicine, Hershey, PA, United States

**Keywords:** IGF2BP1, neuroblastoma, RBP, sensitivity to chemotherapeutics, cell proliferation, CDK inhibitor

## Abstract

Gain at chromosome 17q21 in neuroblastoma is associated with a poor prognosis, independent of MYCN amplification status. Several potential proto-oncogenes have been identified in this region, one of them—insulin-like growth-factor-2 mRNA binding protein (IGF2BP1)—is expressed at high levels in stage 4 tumors, and associated with overall lower patient survival. Here, we demonstrate that down-regulation of IGF2BP1 activity, either by transcript silencing or chemical inhibition, suppresses neuroblastoma cell growth. Furthermore, the combination of IGF2BP1 inhibition along with commonly used chemotherapeutics that broadly affect DNA synthesis, or cyclin-dependent kinase (CDK) inhibitors that disrupt signal transduction, have a synergistic effect on the suppression of neuroblastoma cell proliferation.

## Introduction

Neuroblastoma is an embryonal tumor of the autonomic nervous system, arising from cells within neural-crest tissues (1). Neuroblastoma generally occurs in young children, with the median age at diagnosis is 17 months (2), it is the most common cancer diagnosed during the first year of life (3). The prognosis for neuroblastoma varies significantly, from tumors that regress, to those that become metastatic and resistant to therapy, often resulting in high mortality [reviewed by (4)]. Depending on stage and severity, treatment ranges from surgical resectioning for asystematic low-risk patients, to multiple approach strategies that include surgery, response-adjusted chemotherapy, hematopoietic stem cell transplantation, and local radiation, followed by immunotherapy for high-risk patients (5).

Deregulation of the neural crest and the subsequent development of neuroblastoma has been linked to significant chromosomal alterations that are often associated with poor outcomes [reviewed by (6)]. Neuroblastoma tumorigenesis has long been associated with MYCN amplification at 2p24 (7). A paralog of MYC, MYCN has a similar structure and pro-oncogenic characteristics (8). Like other members of the MYC family, MYCN plays multiple roles in malignancy and maintenance of stem-like state, involved in metastasis, survival, proliferation, pluripotency, self-renewal, and angiogenesis [reviewed by (9)]. Though only found in 25% of neuroblastoma cases, MYCN amplification has been associated with poor prognosis and has remained one of the few genetic prognostic indicators of high-risk neuroblastoma (7).

Larger-scale genetic alterations including chromosomal rearrangements, amplifications and deletions are a more common feature of neuroblastoma cells, with the foremost being the amplification of the 17q chromosomal arm, found in over half of tumors (9, 10). However, it remains unclear which specific oncogenes located in this region contribute to neuroblastoma pathogenesis (11). One of the most promising candidates located in the frequently amplified 17q21 region is the insulin-like growth factor-2 mRNA-binding protein 1 (*IGF2BP1*), a member of the IGF2 mRNA-binding protein family. The *IGF2BP* family function as post-transcriptional regulators and play important roles in transcript regulation, modulating mRNA localization, translation, and stability of various mRNAs during embryogenesis (12).

As an oncogene, IGF2BP1 is known to regulate mRNA transcripts that control tumor cell proliferation, invasion, and chemoresistance [reviewed in (13)] and its elevated expression is associated with poor prognosis in multiple malignancies [reviewed in (12)]. The mRNA targets of IGF2BP1 include numerous tumor-promoting genes, including *c-Myc*,* βTrCP1*, *GLI1*, *MITF*, and *MDR1* (14–18), and has shown to play a critical role in several cancers including: colorectal cancer, melanoma leukemia, basal cell carcinoma, cutaneous squamous cell carcinoma (19–23). In neuroblastoma, IGF2BP1 was shown to be expressed at high levels in stage 4 tumors, and associated with overall lower patient survival (24). Though knockdown or inhibition of IGF2BP1 has been shown to sensitize melanoma to some chemotherapeutic agents and targeted therapy (25–27), its role as a potential therapeutic target in treating neuroblastoma has not been investigated.

In this study, we demonstrate that down-regulation of IGF2BP1 activity, either by transcript silencing or chemical inhibition, suppresses neuroblastoma cell growth. Furthermore, we demonstrate that the combination of IGF2BP1 inhibition along with commonly used chemotherapeutics that broadly effect DNA synthesis, or cyclin dependent kinase (CDK) inhibitors that disrupt signal transduction, have a synergistic effect on suppressing neuroblastoma cell proliferation.

## Materials and Methods

### Cell Line Selection and Culture

The human neuroblastoma cell lines SK-N-AS, SK-N-SH, SK-N-BE (2), and SK-N-DZ were obtained from ATCC (ID#: CRL-2137, HTB-11, CRL-2271, and CRL-2149), and tested for mycoplasma contamination with MycoAlert plus mycoplasma detection kit (Lonza). All cell lines were maintained as monolayers in Dulbecco’s modified Eagle’s medium (DMEM; VWR International), supplemented with 10% v/v fetal bovine serum (FBS, Gibco by Life Technologies) and 100 units/ml penicillin and streptomycin (Corning).

### Generation of SK-N-AS Cells With Inducible Knockdown of IGF2BP1

IGF2BP1 was knocked down using doxycycline inducible SMARTvector Inducible TurboGFP shRNA Lentiviral constructs (Cat. No. V3SH11252- 227953809 GE Dharmacon) shRNA SMARTvector Inducible Non-targeting Control construct (VSC11653 GE Dharmacon). Lentivirus particles were produced from above plasmids in Lenti-X 293T cells (Takara) and were used to transduce SK-N-AS cells to make stable cell lines. Cells were then subjected to 7 days of puromycin selection (1 μg/ml) in growth media prior to induction of knockdown shRNA with doxycycline (1–2 μg/ml) for 3–6 days. Induction of *shIGF2BP1* in SK-N-AS cells showed a depletion of IGF2BP1 by approximately 45–50% (**Supplementary Figure S2**).

### Generation of Human Neuroblastoma Cells With Overexpression of IGF2BP1

GFP-tagged IGF2BP1 overexpression and vector control cell lines were created in SK-N-AS cell line using **LV-GFP-hIMP1 (pLKO. hIMP1_3G from Dr. Joel Yisraeli Lab)** and **EGFP-pReceiver LV105 (Cat. No. EX-GFP-Lv105-B Genecopoeia)** constructs, respectively. Lentivirus particles were produced in 293T cells using lipofectamine 3000 transfection reagent (Invitrogen). Wild-type SK-N-AS cells were transduced by 8-h infections with the lentivirus + 8 ug/ml polybrene reagent. For the genetic rescue experiments the SKNAS cell lines with construct combinations that include: shIGF2BP1 and GFP empty vector control, shIGF2BP1 #1 and GFP-IGF2BP1, shScramble and GFP empty vector control, and shScramble and GFP-IGF2BP1 #1. Puromycin selected shIGF2BP1 and shScramble cells (SK-N-AS) were transduced with the same above-mentioned lentivirus for ectopic expression of IGF2BP1 and the respective GFP control.

### Chemical Compounds and Chemotherapeutics

For the chemical compounds used in this study, refer to **Supplementary Table S1**.

### Protein Isolation and Western Blot Analysis

Cells were washed once with PBS, scraped, collected, and pelleted, following a brief high-speed spin. Excess PBS was removed, and cells were lysed for 20 min on ice in RIPA buffer (100 mM Tris-HCl (pH 7.4), 150 mM NaCl, 1% sodium deoxycholic acid, 1% Triton-X100, and 0.1% SDS), containing the cOmplete™, EDTA-free Protease Inhibitor CocktailEDTA-free Complete protease inhibitors cocktail (Roche). Lysates were centrifuged at 14,000 rpm for 20 min at 4°C, and clarified supernatants collected. Protein concentration was determined with the RC DC™ Protein Assay (Bio-Rad Laboratories). Proteins (50 μg) were separated in 10% polyacrylamide/SDS gels at 100 V, and transferred to PVDF membranes (Millipore), at 100 V at 4°C for 1 h. Membranes were first blocked for 1 h at room temperature with 5% milk powder in PBS containing 0.1% Tween-20 (PBS/T), and then incubated with primary antibodies overnight at 4°C. Primary antibodies include rabbit anti-IMP1 (1:500, Cell Signaling Technologies 8482), rabbit anti-N-Myc (1:1,000, Cell Signaling Technologies 84406), and rabbit anti-β-Actin (1:2,000, Cell Signaling Technologies 4970). Membranes were washed with PBS/T and incubated with HRP-conjugated secondary antibodies (Goat anti-Rabbit IgG; Cell Signaling Technologies 7074) at 1:10,000 for 1 h at room temperature. Proteins were detected with WesternSure Premium Chemiluminescent Substrate on a C-DiGit Blot Scanner (LI-COR Biosciences) and protein bands were quantitated using Image-J software.

### Reverse-Transcription Quantitative Polymerase Chain Reaction (qRT-PCR)

Total RNA was isolated from cells using the RNeasy Mini Kit (Qiagen) according to the manufacturer’s instructions. cDNA was generated using iScript™ cDNA Synthesis Kit (Bio-Rad Laboratories), following the manufacturer’s protocol. cDNA was subjected to real-time PCR using iTaqTM Universal SYBR^®^ Green Supermix (Bio-Rad Laboratories) on a CFX96 Touch™ Real-Time PCR Detection System using primers targeting IGF2BP1 - qHsaCID001074 and RPS18 - qHsaCED0037454 (Bio-Rad Laboratories). The analysis was performed on each sample in triplicate. Relative transcript levels were calculated using the comparative Ct method and normalized to the housekeeping gene RPS18.

### RNA-Seq and Analysis

Three biological replicates of control and IGF2BP1 knockdown cells were collected and RNA was isolated using RNeasy Mini Kit (Qiagen) before being subjected to total RNA sequencing (RNAseq). RNA-seq libraries were generated using KAPA RNA HyperPrep Kits with RiboErase (HMR) (Roche), which targets and depletes rRNA using DNA probes and RNase H. The unique dual index sequences (NEXTFLEX^®^ Unique Dual Index Barcodes, BioO Scientific) were incorporated in the adaptors for multiplexed high-throughput sequencing. The final product was assessed for its size distribution and concentration using BioAnalyzer High Sensitivity DNA Kit (Agilent Technologies). The libraries were pooled and diluted to 3 nM using 10 mM Tris-HCl, pH 8.5 and then denatured using the Illumina protocol. The denatured libraries were loaded onto an S1 flow cell on an Illumina NovaSeq 6000 (Illumina) and run for 2X50 cycles according to the manufacturer’s instructions. De-multiplexed and adapter-trimmed sequencing reads were generated using Illumina bcl2fastq (released version 2.18.0.12) allowing no mismatches in the index read. BBDuk (http://jgi.doe.gov/data-and-tools/bb-tools/) was used to trim/filter low-quality sequences using “qtrim=lr trimq=10 maq=10” option. Next, alignment of the filtered reads to the human reference genome (GRCh38) was done using HISAT2 (version 2.1.0) (28) applying –no-mixed and –no-discordant options. Read counts were calculated using HTSeq (29) by supplementing Ensembl gene annotation (GRCh38.78). EdgeR (30) was used to fit the read counts to the negative binomial model along with generalized linear model (GLM) and differentially expressed genes were determined by the likelihood ratio test method implemented in the edgeR package. Significance was defined to be those with q-value <0.01 calculated by the Benjamini-Hochberg method to control the false discovery rate (FDR) and log2 fold change is greater than 1 or smaller than −1. The list of differentially expressed genes was analyzed with Ingenuity Pathway Analysis. Ingenuity Pathway Analysis (Qiagen) was used to analyze the RNA-seq results and generate possible pathways/genes affected by knocking down IGF2BP1.

### IncuCyte Cell Proliferation Assay

The knockdown shRNA and IGF2BP1 overexpression cell lines were induced with 1–6 μg/ml doxycycline for 48 h before they were trypsinized and seeded at 1 × 10^4^/well in a 96-well plate. Parental SK-N-AS, SK-N-BE(2), and SK-N-DZ cells were seeded at 2 × 10^4^/well in a 96-well plate. After 24 h, the media was replaced with fresh media containing DMSO or chemicals at the indicated concentrations, and cells were placed in the IncuCyte Live-Cell Analysis System (Satorious). Live-cell phase contrast images were obtained using a 10× objective lens (three images per well) within the instrument, and cell density was analyzed using IncuCyte Live Cell Analysis (v2019B) software.

### IncuCyte Apoptosis Assay

Parental SK-N-AS, SK-N-BE(2), and SK-N-DZ cells were seeded at 3 × 10^5^/well in a 96-well plate. After 24 h, the media was aspirated and replaced with fresh media containing Annexin V Red Reagent as per the manufacturer’s instructions (Sartorious Cat. No. 4641). In each experimental condition, DMSO and chemicals were added to the media at the indicated concentrations and cells were placed in the IncuCyte Live-Cell Analysis System (Satorious). Live-cell images were obtained using a 10× objective lens capturing phase contrast and red NIR (three images per well) within the instrument. An apoptotic index was calculated with the IncuCyte Cell-by-Cell Analysis Software Module (Sartorious Cat. No. 9600-0031).

### Statistical Analysis

SK-N-AS, SK-N-BE(2), and SK-N-DZ neuroblastoma cells were collected and studied with regression analysis. Seven drugs, Cyclophosphamide, Vincristine, Doxorubicin, Etoposide, Topotecan, Dinaciclib, and AZD4573 were included in the study and compared with the control group DMSO and the IGF2BP1 inhibitor BTYNB. Three independent measurements on confluency percentages were recorded under DMSO, BTYNB, the selected inhibitor and the combination of the inhibitor and BTYNB respectively over seven time points for 144 h.

To model the cell growth curves under each condition, a linear regression model was fit to this dataset:

$$y_{confluency,i}=\beta_{0}+\beta_{1}x_{time,i}+\beta_{2}x_{Inhibitor,i}+\beta_{3}x_{BTNYNB,i}+\beta_{4}x_{Inhibitor,i}*x_{BTNYNB,i}*+\beta_{5}x_{time,i}*x_{Inhibitor,i}+\beta_{6}x_{time,i}*x_{BTYNB,i}+\beta_{7}x_{time,i}*x_{Inhibitor,i}*x_{BTNYNB,i}+\epsilon_{i}$$

Where i = 1,2,…,28 and ϵ*_i_* was the error term. *x_Inhibitor,i_* and *x_BTYNB,i_* were the indicators of whether using inhibitor, BTYNB, control or both inhibitor and BTYNB. The parameters β_5_ and β_6_ were the rate of change in confluency percentage with inhibitor or BTYNB compared to that with control group DMSO. The parameter β_7_ further investigated the additional effect on growth rate under the combination of the specific inhibitor and BTYNB.

## Results

### Depletion of IGF2BP1 Reduces the Rate of Cell Proliferation in Neuroblastoma Cells

To elucidate the contribution of IGF2BP1 to protumorigenic phenotype of both low- and high-risk neuroblastoma cells, we employed three cell lines with varying expression of two genetic markers associated with poor prognosis, *MYCN* and *IGF2BP1*. The SK-N-AS cell line represents low-risk neuroblastoma, with barely detectable levels of MYCN and IG2BP1 expression. SK-N-BE(2) cells represent intermediate-risk neuroblastoma, with moderate expression of MYCN and low expression of IGF2BP1. The SK-N-DZ cell line represents high-risk neuroblastoma, with abundant expression of MYCN and IGF2BP1 (**Supplementary Figure S1**).

**Figure 1:**
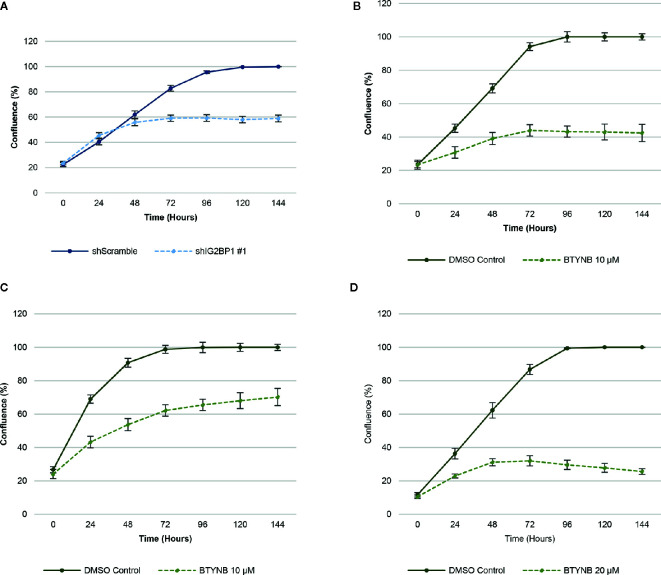
Down-regulation of IGF2BP1 activity decreases human neuroblastoma cell proliferation. Cell proliferation assays of neuroblastoma cell lines [SK-N-AS, SK-N-BE(2), and SK-N-DZ] were determined by live-cell phase contrast. **(A)** Depletion of IGF2BP1 mRNA was achieved by induction of SK-N-AS cells containing shScramble or shIGF2BP1 constructs. Cells were induced with doxycyline (1 μg/ml) for 72 h prior to the imaging. **(B)** SK-N-AS and **(C)** SK-N-BE(2) cells were treated with 10 μM BTYNB and **(D)** SK-N-DZ cells were treated with 20 μM BTYNB along with a DMSO vehicle control. Cell confluence was measured by live cell phase contrast imaging over 6 days, n = 3.

In order to ensure efficient depletion of IGF2BP1, we have developed a lentiviral system with inducible IGF2BP1 knockdown in the SK-N-AS cells. The inducible system was chosen to avoid long-term consequences of possible selection pressure in cells where IGF2BP1 function is inhibited for multiple passages. Inducible IGF2BP1-specific shRNA reduced cell proliferation by 40–45% over a 6-day period in SK-N-AS cells (**Figure 1**). Treatment with BTYNB, an inhibitor of IGF2BP1 RNA binding (27), had a similar effect on cell proliferation. At 10 μM BTYNB, cell proliferation was decreased in SK-N-AS by 60% and by 35–40% in SK-N-BE(2) cells, respectively. Twice the dose of BTYNB (20 μM) was required to elicit a similar effect (35–40% decrease in cell proliferation) on the SK-N-DZ cells (**Figure 1**).

To confirm the specificity of our results, we created co-transduced SK-N-AS cells constitutively expressing GFP empty vector and GFP-IGF2BP1 constructs in cells containing doxycycline inducible shScramble and shIGF2BP1 #1 RNAs. Upon induction, SK-N-AS cells depleted of IGF2BP1 with the GFP empty vector had a cell proliferation rate that was reduced by up to 90%, and the effect of this knockdown was completely reversed by ectopic expression of GFP-tagged IGF2BP1 (**Supplementary Figure S5**). Similar effects were observed in the SK-N-BE(2) cell lines (data not shown). A similar trend was observed when SK-N-AS cells expressing GFP constructs were treated with 4 μM BTYNB (**Supplementary Figure S5**). After 60 h in the cell proliferation assay, BTYNB treated cells with GFP empty vector was reduced by 30%, while those expressing the GFP-IGF2BP1 was reduced by only 20%. Conversely, when SK-N-AS cells expressing GFP-IG2BP1 and DMSO, cell proliferation was increased by 10% compared to cells expressing the GFP empty vector (**Supplementary Figure S5**). Although statistically significant, the rescue of cell proliferation with over-expression of IGF2BP1 was less effective in BTYNB-treated cells. This could be due to the nature of chemical inhibition that is less dependent on the levels of IGF2BP1. Although non-specific effects of BTYNB on cell proliferation through its effect on other IGF2BP1 paralogues or other effector molecules cannot be ruled out. Taken together, these findings indicate that depletion and/or inhibition of IGF2BP1 result in decreasing cell replication, and show high specificity of IGF2BP1 knockdown in observed inhibition of proliferation.

### Effect of IGF2BP1 Inhibition on the Transcriptome of Neuroblastoma Cells

**Figure 2:**
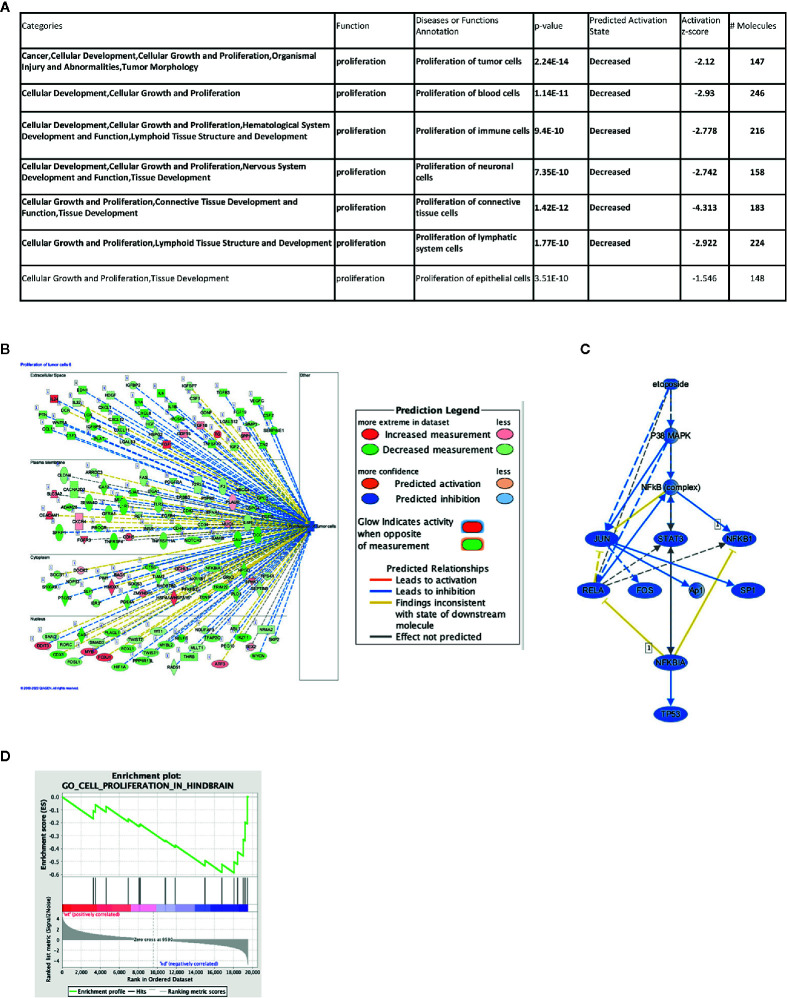
Impact of IGF2BP1 depletion on the neuroblastoma transcriptome. SK-N-AS cell lines expressing shScramble or shIGF2BP1 constructs were subjected to RNAseq (n = 3 per cell line) and differentially expressed gene signatures were examined. **(A, B)** Representative significantly altered categories in diseases and functions based on Ingenuity Pathway Analysis are shown. **(A)** Seven categories were significantly enriched that are classified as “proliferation” function. Six of them (highlighted in bold) showed decreasing state by IGF2BP1 depletion. **(B)** The top-ranked category (Proliferation of tumor cells) is visualized by a subcellular network. **(C)** A mechanistic network was built around one of the predicted upstream regulators, Etoposide, a commonly used chemotherapeutic agent used to treat neuroblastoma. **(B, C)** Observed gene expression measurement and activation states are represented in colored oval objects as illustrated in the legend. The solid lines represent direct interactions and the dashed lines represent indirect interactions. **(D)** Gene Set Enrichment Analysis in IGF2BP1 depleted SK-N-AS in cell proliferation in hindbrain.

One of the major functions of IGF2BP1 is the modulation of RNA stability and, therefore, abundance of its target RNAs. To understand possible mechanisms of how the depletion of IGF2BP1 impacts cell proliferation, we performed total RNAseq on SK-N-AS cells upon IGF2BP1 knockdown. A total of 3,581 genes were significantly differentially expressed with IGF2BP1 depletion (**Supplementary Table S2**) and subjected to Ingenuity Pathway Analysis (IPA, Qiagen), Gene Set Enrichment Analysis (GSEA), and gene expression network analysis. Our results show that broadly, the majority of transcripts that were adversely affected by IGF2BP1 depletion were associated with cancer, organismal injury and abnormalities, several disease areas including gastrointestinal, endocrine system, dermatology, reproductive systems, and cellular movement. Importantly, IGF2BP1 depletion resulted in inhibitory effect in categories enriched with transcripts associated with proliferative functions (**Figures 2A, B**, **Supplementary Figure S3**, and **Supplementary Table S3**). GSEA analysis showed pathways associated with hindbrain cell proliferation and transcripts associated with cell cycle regulation were significantly enriched in IGF2BP1 depleted cells (**Figure 2D** and **Supplementary Figure S4**). Interestingly, Upstream Regulator Analysis (URA), which determines likely upstream regulators that are connected to dataset genes through a set of direct or indirect relationships, manifested several commonly used chemotherapeutic agents used to treat neuroblastoma, including Cisplatin, Cyclophosphamide, Doxorubicin, Etoposide, and Topotecan (**Figure 2C** and **Supplementary Figure S4**). A mechanistic network built around each drug suggested the involvement of overlapping downstream targets, including the tumor protein p53 (TP53), phosphoinositide-3-kinase–protein kinase B/Akt (PI3K-PKB/Akt), the extracellular signal-regulated protein kinases 1 and 2 (ERK1/2), the composite transcription factor activating protein (c-Jun/AP-1) and Signal transducer and activator of transcription 3 (STAT3). Taken together, these findings suggest that many cancer-related proliferation-promoting pathways are altered by IGF2BP1 depletion in neuroblastoma. Furthermore, the pathways suppressed by standard chemotherapeutics are similarly suppressed by the IGF2BP1 depletion, providing a mechanistic rationale of the potential therapeutic application of IGF2BP1.

### Inhibition of IGF2BP1 Sensitizes Neuroblastoma Cells to Chemotherapeutics

Given our results on the depletion of IGF2BP1 in neuroblastoma cell cycle and the identification of multiple gene networks associated with common chemotherapeutics that target DNA synthesis and cell division, we designed cell proliferation assays to measure the combinatorial effect of IGF2BP1 inhibition with multiple chemotherapeutic agents used to treat neuroblastoma patients.

All the doses used in our study are below or within the range of typically achieved plasma concentration during the course of chemotherapy which is 0–100 µM, 0–100 nM, 0–1 µM, 0–20 µM, and 0–200 nM for Cyclophosphamide, Vincristine, Doxorubicin, Etoposide, and Topotecan respectively.

**Figure 3:**
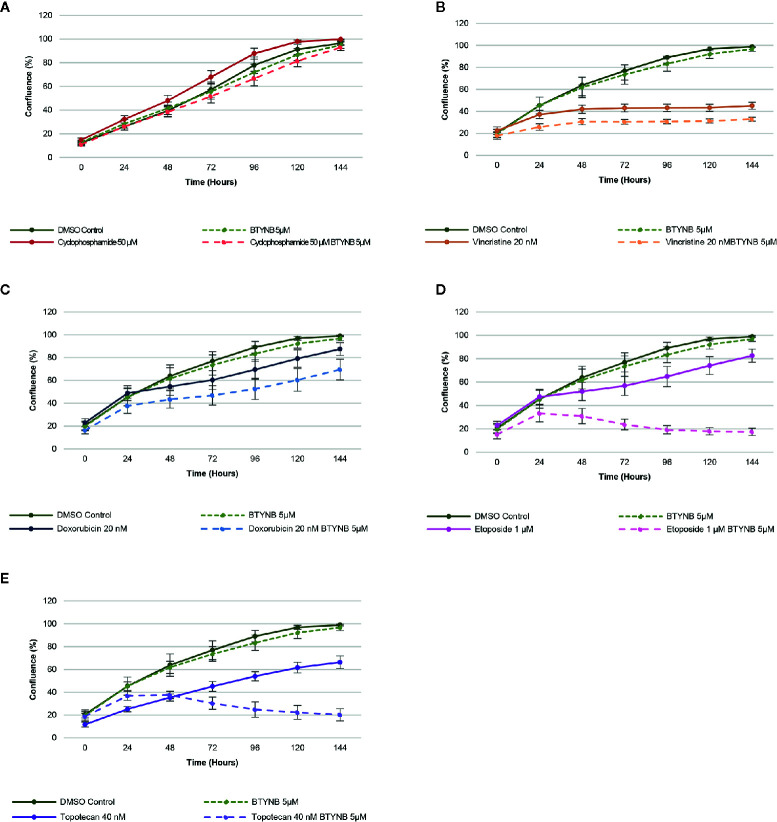
IGF2BP1 inhibition sensitizes SK-N-AS cells to chemotherapeutic agents. Comparison of the rate of growth of SK-N-AS cells with BTYNB combined with multiple chemotherapeutic agents, including **(A)** Cyclophosphamide, **(B)** Vincristine, **(C)** Doxorubicin, **(D)** Etoposide, and **(E)** Topotecan. Each drug was tested separately and in combination as indicated and cell confluence was compared to the DMSO control over an interval of 6 days, n = 3.

When treated with a lower concentration of BTYNB (5 μM), cell proliferation of SK-N-AS cells were not significantly diminished when compared to the DMSO control; however, when combined with another chemotherapeutic agent, cell proliferation was often reduced further than the chemotherapeutic agent alone (**Figure 3**). Cyclophosphamide had no detectable effect on cell proliferation, while the addition of Vincristine alone reduced observed proliferation rates to 40% in SK-N-AS, 55% in SK-N-BE(2), and 40% in SK-N-DZ compared to DMSO controls (**Figure 3** and **Supplementary Figure S6** and **S7**). In combination with BTYNB, proliferation rates dropped an additional 5–10%. Doxorubicin reduced cell proliferation to 80% in SK-N-AS, ~55% in SK-N-BE(2), and ~50% in SK-N-DZ compared to controls, and was reduced a further 5–20% when combined with BTYNB (**Figure 3** and **Supplementary Figure S6** and **S7**). Regression analysis revealed that the effects observed between BTYNB and Etoposide were synergistic in SK-N-AS and SK-N-BE(2). Synergy was also detected when treated with BTYNB and Topotecan in SK-N-AS, SK-N-BE(2), and SK-N-DZ (**Supplementary Table S4**). It was notable that our analysis indicated that the combinations that included Vincristine, Doxorubicin, and Etoposide had adverse effect, in which the rate of proliferation of any single drug was lower than when it was combined with BTYNB (**Supplementary Table S4**). These results suggest that the combination of IGF2BP1 inhibition has an additive, and in some cases, a synergistic effect in cells representing low- and intermediate-risk neuroblastoma.

### Inhibition of IGF2BP1 Sensitizes Neuroblastoma to CDK Inhibitors

**Figure 4:**
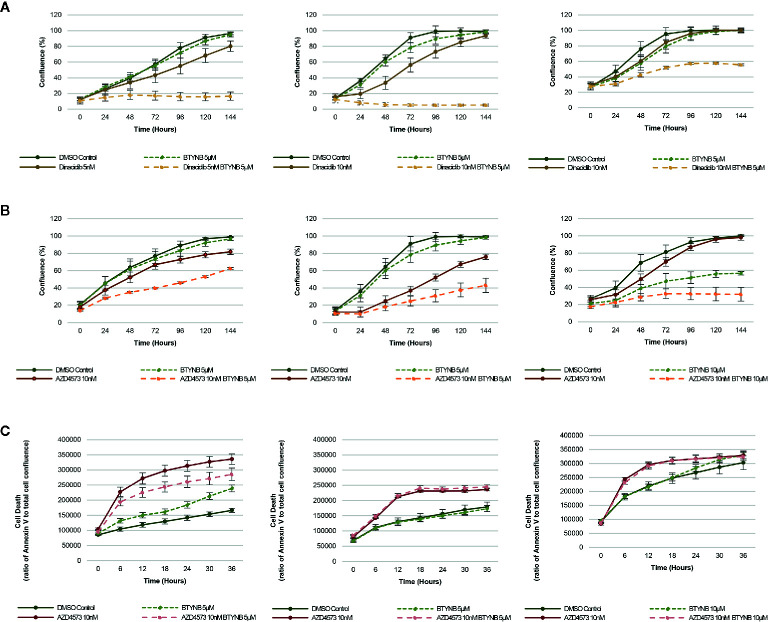
IGF2BP1 inhibition sensitizes neuroblastoma to CDK inhibitors. Comparison of the rate of growth of neuroblastoma cells with **(A)** BTYNB and Dinaciclib, and **(B)** BTYNB and AZD4573. Drugs were tested separately and combined at the concentrations indicated and cell confluence was compared to the DMSO control over an interval of 6 days, n = 3. **(C)** Apoptosis assay combining BTYNB and AZD4573 in human neuroblastoma cell lines. Cell death was estimated as a ratio of Annexin V red signal to total cell confluence. In each experimental condition measurements were recorded in 6 h intervals for 36 h, n = 3.

In addition to chemotherapeutic agents more commonly used to treat neuroblastoma, we sought to determine if IGF2BP1 inhibition could be combined with more potent inhibitors of cell proliferation. Drug toxicity and resistance remains one of the most significant barriers to the delivery of curative doses of cancer chemotherapy, therefore, any strategy that could be used to treat neuroblastoma patients with lower doses would be advantageous. To test this hypothesis, we set up cell proliferation assays combining the IGF2BP1 inhibitor BTYNB with Dinaciclib. a potent, multiple- cyclin-dependent kinase (CDK) inhibitor has been shown to suppress neuroblastoma growth by inhibiting CDK2 and CDK9 activity (31). At 5nM, Dinaciclib reduced cell proliferation only by 20% in SK-N-AS cells, but when combined with 5 μM BTYNB, cell proliferation fell to less than 80% compared to the DMSO control (**Figure 4A**). In SK-N-BE(2) cells, there was only a moderate decrease in proliferation of 20–30% with 10 nM Dinaciclib, but when combined with 5 μM BTYNB, there was a 90% reduction (**Figure 4A**). Though there was no substantial decrease in cell proliferation in SK-N-DZcells with Dinaciclib alone, the combination of 10 nM Dinaciclib and 5 μM BTYNB reduced cell proliferation by 40% (**Figure 4A**). Regression analysis showed a synergistic effect between Dinaiclib and BTYNB in SK-N-AS and SK-N-BE(2), but not in SK-N-DZ cells (**Supplementary Table S5**).

Next, we tested the combination of BTYNB with AZD4573, a highly selective CDK9 inhibitor that has been shown to arrest cell proliferation and induce apoptosis in hematologic cancers (32). Addition of 10 nM AZD4573 reduced cell proliferation by 20% in SK-N-AS cells, and when combined with 5 μM BTYNB they reduced cell proliferation by 40% compared to the DMSO control (**Figure 4B**). In SK-N-BE(2) cells, 10 nM AZD4573 reduced cell proliferation by 25% alone and by 60% when combined with 5 μM BTYNB compared to the DMSO control (**Figure 4B**). In SK-N-DZ cells, 10 nM AZD4573 only had a marginal effect reducing cell proliferation at earlier time points by 10–20%; however, when combine with 10 μM BTYNB cell proliferation was reduced to 70% of the controls (**Figure 4B**). Regression analysis showed a synergistic effect between AZD4573 and BTYNB in SK-N-AS, SK-N-BE(2), and SK-N-DZ (**Supplementary Table S5**).

Because the known effect of AZD4573 to decrease cell viability, we performed apoptotic assays to measure the levels of Annexin V over a 36 h period and found that though AZD4573 did increase cell toxicity in SK-N-BE(2) and SK-N-DZ, it was not further increase with the addition of BTYNB, and in SK-N-AS, it appears the addition of BTYNB may have decreased the cytotoxic effect of AZD4573 (**Figure 4C**).

## Discussion

Previous work has demonstrated that knocking down or inhibiting IGF2BP1 in some cancers such as melanoma sensitizes the cells to chemotherapeutic and targeted agents (25–27). With the amplification of the 17q chromosomal arm—including the *IGF2BP1* gene locus—being one of the most common genetic alterations in neuroblastoma, and the linkage of IGF2BP1 expression serving as a prognosticator of poor prognosis (12, 24), we present evidence to support that IGF2BP1 is a promising novel therapeutic target for neuroblastoma.

To this aim, we used three different human neuroblastoma cell lines, SK-N-AS, SK-N-BE(2), and SK-N-DZ and employed both short hairpin RNA (shRNA)-mediated inducible knockdown or chemical inhibition of IGF2BP1 mRNA binding to test our hypothesis that suppressing IGF2BP1 activity would have a negative impact on cell growth and proliferation. These cell lines were chosen because of their relative expression of both MYCN and IGF2BP1, with SK-N-AS expressing minimal levels of either proteins and representing low-risk neuroblastoma, SK-N-BE(2) expressing moderate levels and representing intermediate-risk neuroblastoma, and SK-N-DZ expressing high levels and representing high risk neuroblastoma (**Supplementary Figure S1**). Perhaps because of the presumably high copy number and over-expression of IGF2BP1 in SK-N-BE(2) and SK-N-DZ, it was difficult to create inducible shRNA IGF2BP1 cells in either neuroblastoma line. We observed that the SK-N-DZ, and to a lesser extent the SK-N-BE(2) cell lines, were often more resistant to IGF2BP1 inhibition when compared to the SK-N-AS cell line. This was compensated for by increasing the concentration of BTYNB enough to alter SK-N-DZ cell proliferation (**Figures 1**, **3**, **Supplementary Figure S7**, and **Figure 4**) and may reflect the necessity of using dynamic drug dosage when treating more malignant neuroblastomas. It is well established IGF2BP1 binds and protects mRNA of several tumor-promoting genes in multiple cancers (14–23), therefore, it was not surprising to discover many of the transcripts down regulated in IGF2BP1 depleted SK-N-AS cells were associated with cell proliferation and viability, migration, and cancer. Conversely, mRNA transcripts associated with cell mortality and apoptosis were both enhanced, suggesting that IGF2BP1 plays an important role in promoting oncogenesis in neuroblastoma. Perhaps even more interestingly, downstream gene enrichment analysis showed an overlap of gene networks between IGF2BP1 depletion and several common chemotherapeutics including, Cyclophosphamide, Vincristine, Doxorubicin, Etoposide, and Topotecan. The genes associated with these networks included transcripts associated with tumorgenesis and cell proliferation, leading us to believe IGF2BP1 suppression could result in further sensitization of neuroblastoma to these drugs.

Combination IGF2BP1 inhibition with some of the routinely used chemotherapeutics is promising as it can provide better efficiency at lower doses resulting in less severe side effects. This was also a factor when using the IGF2BP1 inhibitor BTYNB, for which 10 µM was sufficient to reduce cell proliferation in low- and intermediate-risk neuroblastoma cell lines [SK-N-AS and SK-N-BE(2)], and 15 µM was needed in the high-risk neuroblastoma cell line (SK-N-DZ), were reduced to 5 and 10 µM, respectively. With the exception of Cyclophosphamide which did not have an effect on cell proliferation *in vitro*, neuroblastoma cell proliferation was not only decreased when treated with significantly lower concentrations of Vincristine, Doxorubicin, Etoposide, and Topotecan, growth rates were further diminished when combined with BTYNB. Perhaps even more interestingly, statistical analysis of combined growth rates showed a synergistic effect between the combinations of BTYNB and Etoposide in SK-N-AS and SK-N-BE(2), and BTYNB and Topotecan drugs in all three neuroblastoma cell lines. This is particularly encouraging because this was achieved using significantly lower concentrations than the maximum dosage of Etoposide (20 µM) and Topotecan (200 nM). Taken together, this suggests that an effective treatment of both low- and high-risk neuroblastomas could employ this dual strategy.

Because of the success we observed in combining IGF2BP1 inhibition with commonly used therapeutics, we endeavored to determine whether we could replicate these results with drugs even more efficient at inhibiting neuroblastoma cell proliferation at lower doses. One potential class of drugs includes CDK inhibitors. Used at low concentrations, Dinaciclib is a broad CDK inhibitor (CDK1, 2, 5, and 9), but its antagonistic effect on CDK2 and CDK9 has been attributed to its ability to suppress neuroblastoma cell proliferation (31). Indeed, we observed a near total cessation of cell proliferation in SK-N-AS and SK-N-BE(2) cell lines when we combined BTYNB and Dinaciclib, and a significant decrease in proliferation in the high-risk SK-N-DZ cell line.

Next we attempted to determine whether CDK2 and CDK9 inhibition, when combined with IGF2BP1 inhibition, would have an additive effect on neuroblastoma cell replication. We were able to rule out CDK2 inhibition with the use of NU6300, a covalent ATP-competitive CDK2 inhibitor, which had no effect on the proliferation of neuroblastoma cell lines that we tested (data not shown). However, when we combined BTYNB with AZD4573, a highly selective CDK9 inhibitor that has recently been shown to induce apoptosis in hematologic cancer (32), we observed a synergistic effect between both inhibitors, significantly decreasing cell proliferation in each neuroblastoma cell line tested. We also tested the effect of this drug combination on cell viability using an Annexin V-based apoptosis assay and confirmed that AZD4573 does decrease cell mortality, but this effect is not enhanced with treatment of BTYNB.

Whether in combination with conventional chemotherapeutic agents, or CDK inhibitors, we propose that the inhibition of IGF2BP1 function represents a promising novel strategy for future neuroblastoma treatment. Once more efficient and well tolerated IGF2BP1 inhibitors are developed, this combinatorial approach could result in higher efficiency and lower drug resistance and toxicity, resulting in better outcome for patients.

## Data Availability Statement

The original contributions presented in the study are publicly available. This data can be found here: https://www.ncbi.nlm.nih.gov/geo/query/acc.cgi?acc=GSE158258.

## Author Contributions

JB, MD, and CG performed the experiments. SG and YK were involved in data analysis. JB, SG, YK, and VS were involved in designing the study and writing the paper. All authors reviewed the results. All authors contributed to the article and approved the submitted version.

## Funding

This study was supported in part by the NIH NCI grants CA191550, CA243167, and the Four Diamonds Fund of the Pennsylvania State University College of Medicine (VS).

## Conflict of Interest

The authors declare that the research was conducted in the absence of any commercial or financial relationships that could be construed as a potential conflict of interest.
