# Supplementary material for: Inhibition of the mRNA-Binding Protein IGF2BP1 Suppresses Proliferation and Sensitizes Neuroblastoma Cells to Chemotherapeutic Agents
Source: Front Oncol. 2021 Mar 16;11:608816. doi: 10.3389/fonc.2021.608816 (PMC8008117; doi:10.3389/fonc.2021.608816)
Supplement: Supplementary file 1 [file DataSheet_1.pdf]

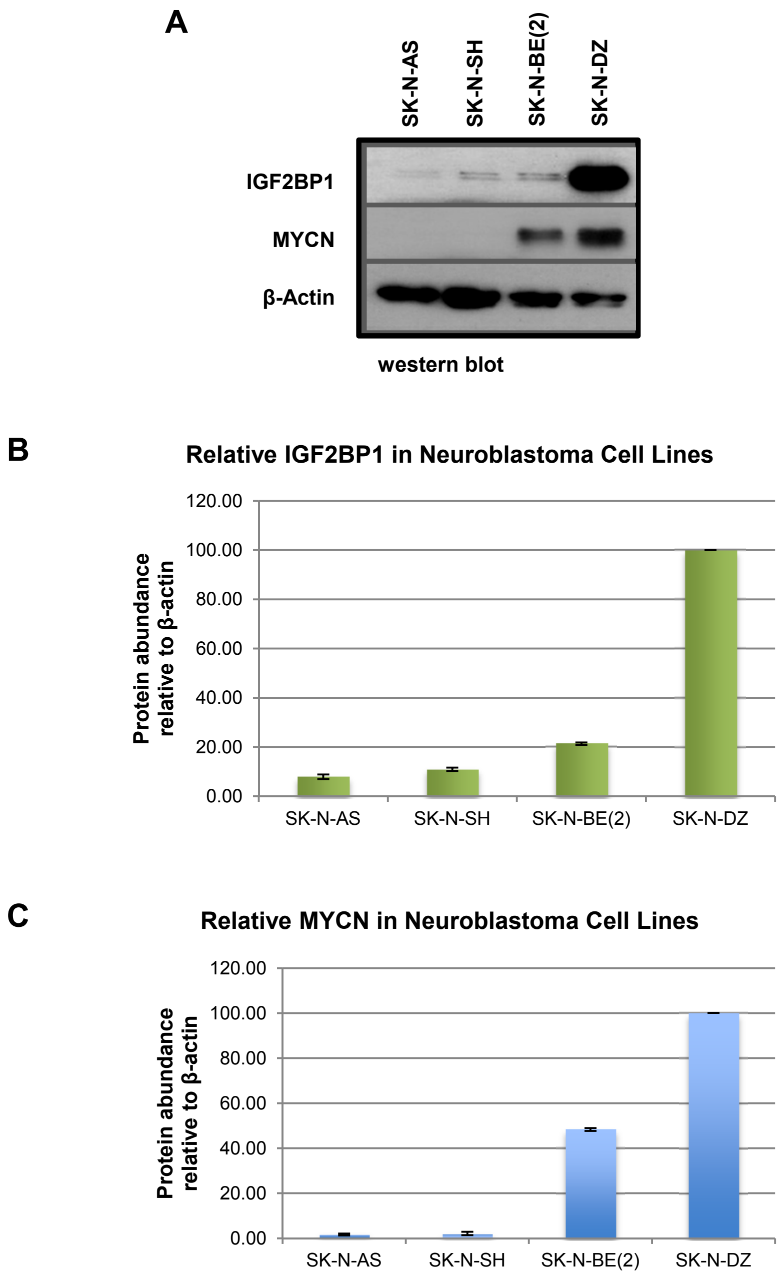

Supplementary Figure S1. Expression of IGF2BP1 and MYCN in human neuroblastoma cells
The prognostic markers, IGF2BP1 and MYCN, were assessed in the neuroblastoma cell lines: SK-N-AS, SK-N-SH, SK-N-BE(2), and SK-N-DZ. (A) The protein level of IGF2BP1 and MYCN were detected by western blot. Wells were loaded with 50 μg total protein lysate and β-Actin served as a loading control. (B) The relative abundance of IGF2BP1 (B) and MYCN (C) were determined by normalizing protein abundance to β-Actin in order to compare between each neuroblastoma cell line.

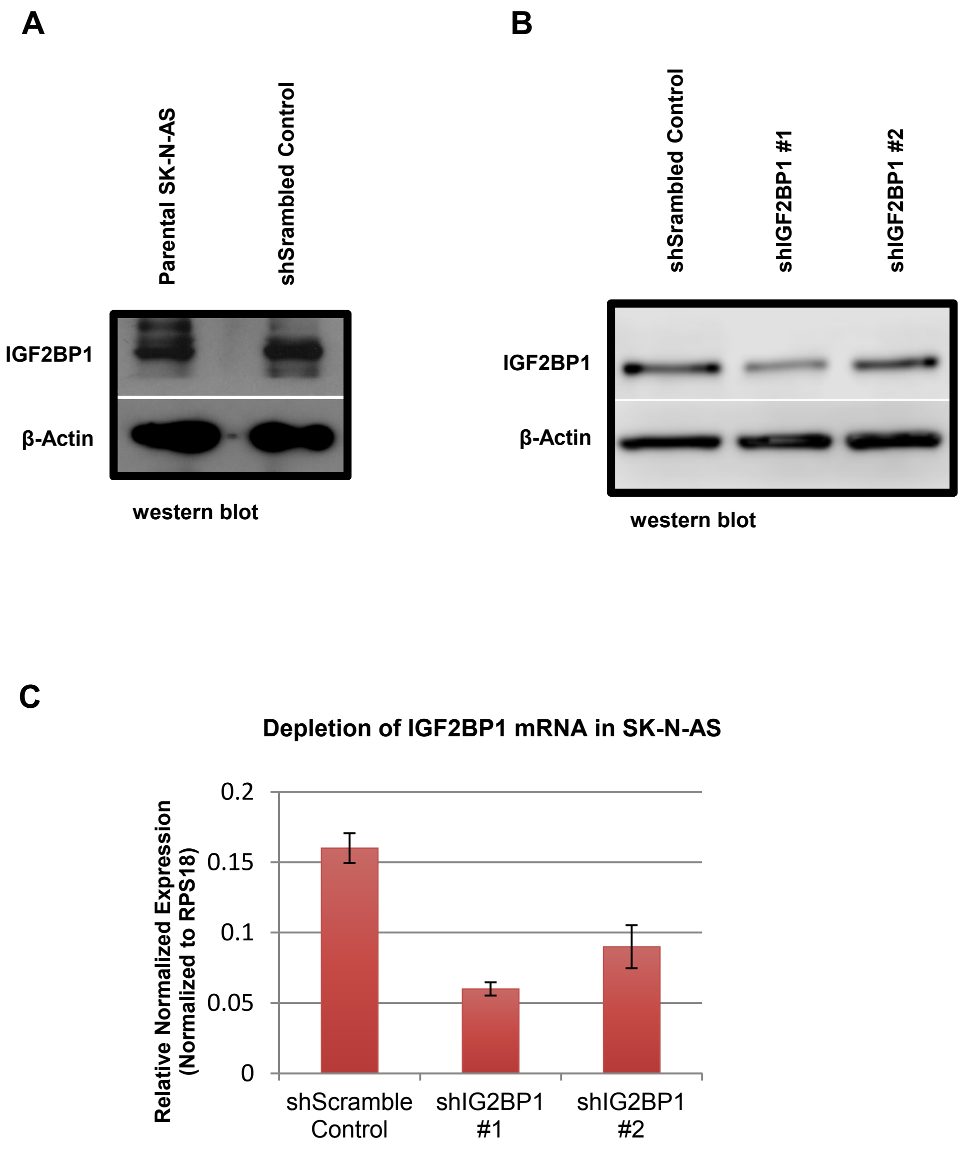

Supplementary Figure S2. Depletion of IGF2BP1 in SK-N-AS cells

A doxycycline inducible lentiviral system was used with a shScramble and two shIGF2BP1 constructs to achieve depletion of IGF2BP2 in SK-N-AS cells. (A) IGF2BP1 expression in shScramble transduced cells relative to that found in the SK-N-AS parental line (treated with doxycycline for 6 days), and (B) depletion of IGF2BP1 by two shIGF2BP1 constructs. The protein level of IGF2BP1 was detected by western blot 6 days after doxycycline induction. Wells were loaded with 50 μg total protein lysate and β-Actin served as a loading control. (C) The mRNA transcript abundance of IGF2BP1 was analyzed from cells in the same induction experiment by real-time quantitative reverse transcription PCR (qRT-PCR) and normalized to RPS18.0

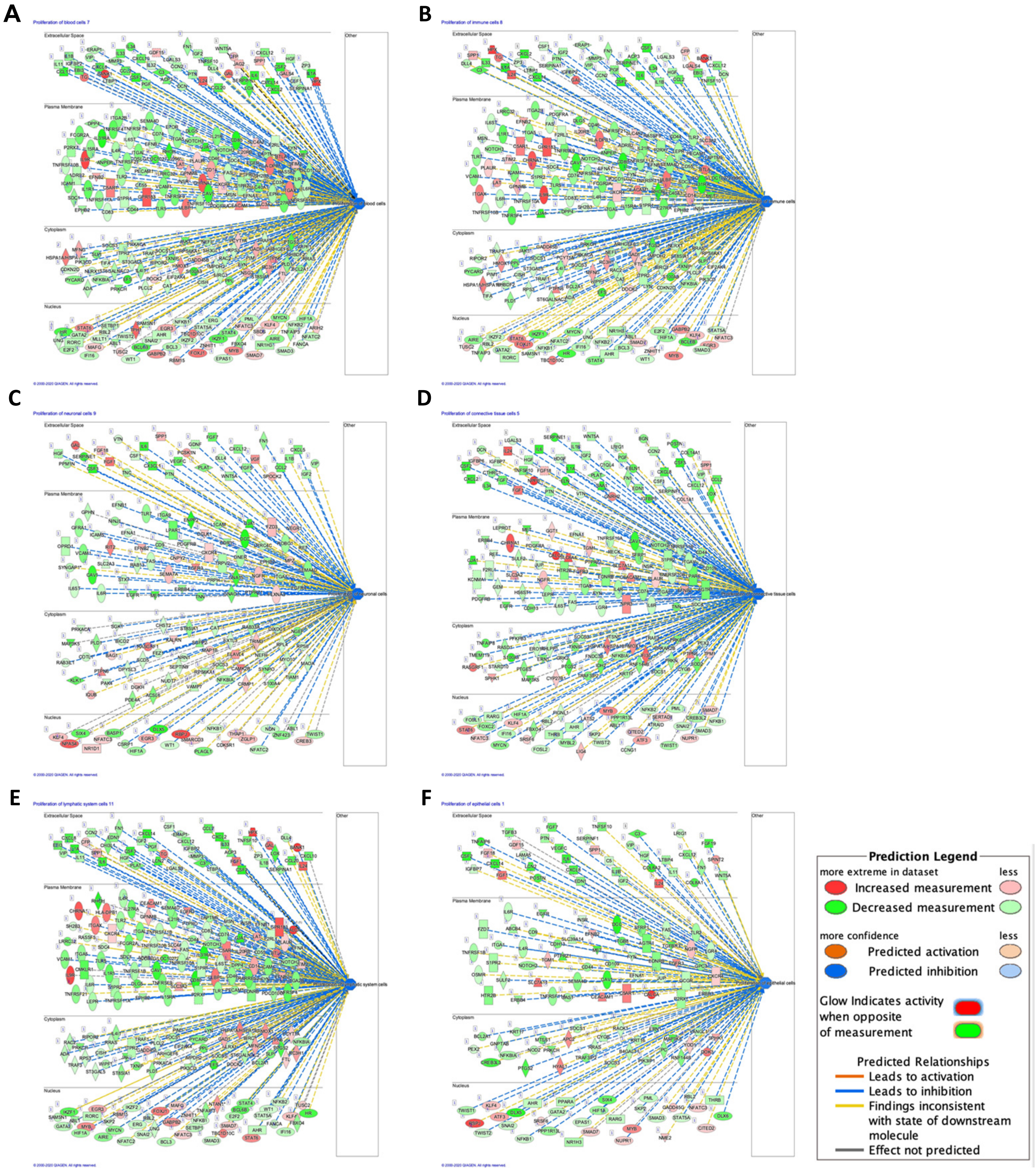

Supplementary Figure S3. Gene enrichment analysis in IGF2BP1 depleted SK-N-AS cells

SK-N-AS cell lines expressing shScramble or shIGF2BP1 constructs were subjected to RNAseq (n=3 per cell line) and differentially expressed gene signatures were examined. (A-F) 6 categories that are significantly altered in diseases and functions based on Ingenuity Pathway Analysis are visualized by subcellular networks. (A) Proliferation of blood cells. (B) Proliferation of immune cells. (C) Proliferation of neuronal cells. (D) Proliferation of connective tissue cells. (E) Proliferation of lymphatic system cells. (F) Proliferation of epithelial cells. (A-F) Observed gene expression measurement and activation states are represented in colored oval objects as illustrated in the legend.

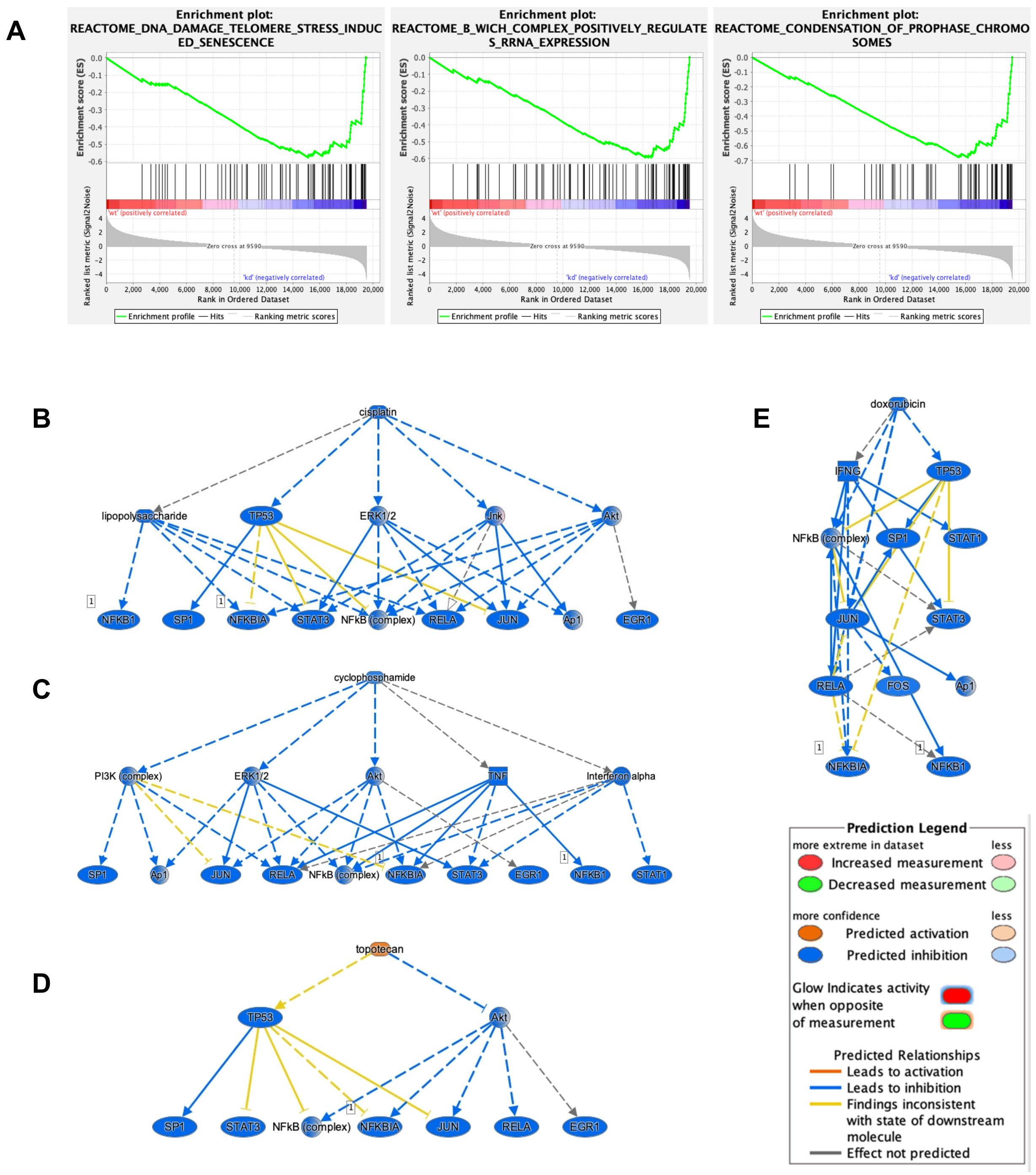

Supplementary Figure S4. Gene enrichment and network analysis in IGF2BP1 depleted SK-N-AS cells

SK-N-AS cells containing doxycycline inducible shScramble and shIGF2BP1 constructs were harvested 6 days after induction, RNA was extracted, and was analyzed by RNAseq. (A) Gene Ontology (GO) enrichment assays revealed several gene groupings that were down-regulated in cell cycle. Gene network analysis showed that multiple mRNA transcripts down-regulated in IGF2BP1 depleted SK-N-AS cells overlapped with networks associated with downstream targets of chemotherapeutic agents including (B) Cisplatin, (C) Cyclophosphamide, (D) Topotecan, and (E) Doxorubicin. (B-E) Observed gene expression measurement and activation states are represented in colored oval objects as illustrated in the legend.

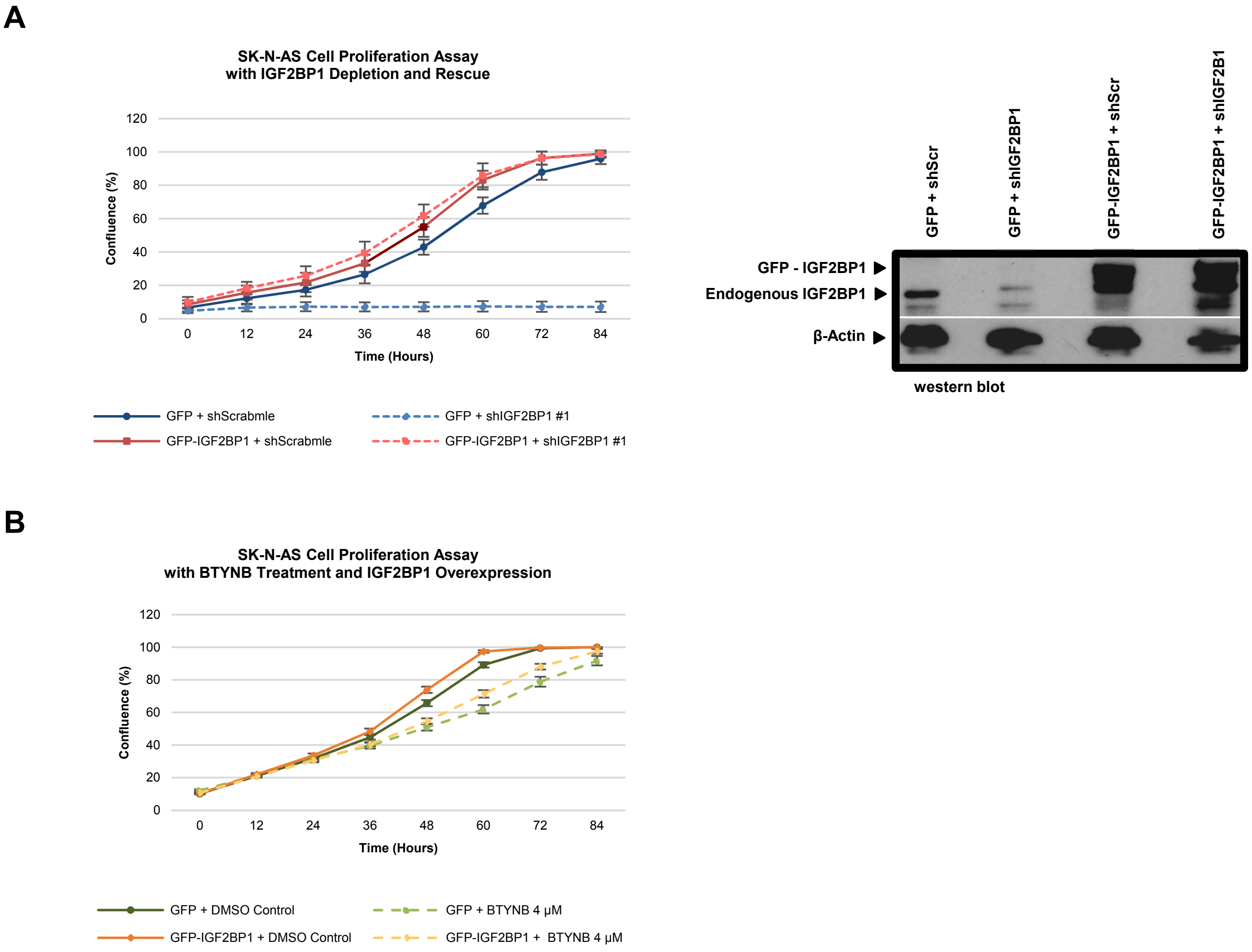

Supplementary Figure S5. IGF2BP1 overexpression rescues SK-N-AS cells from IGF2BP1 depletion and inhibition

(A) Comparison of the the rate of growth of co-transduced SK-N-AS cells expressing GFP empty vector control or GFP-IGF2BP1 and doxycycline inducible shScramble or shIGF2BP1 #1 constructs. Western blot analysis showing the expression of endogenous and ectopically expressed IGF2BP1 shown on the right. (B) Comparison of the rate of growth of SK-N-AS cells containing GFP empty vector or GFP-IGF2BP1 constructs in combination with treatment of 4 μM BTYNB and was compared to the DMSO control over an interval of 6 days, n=3.

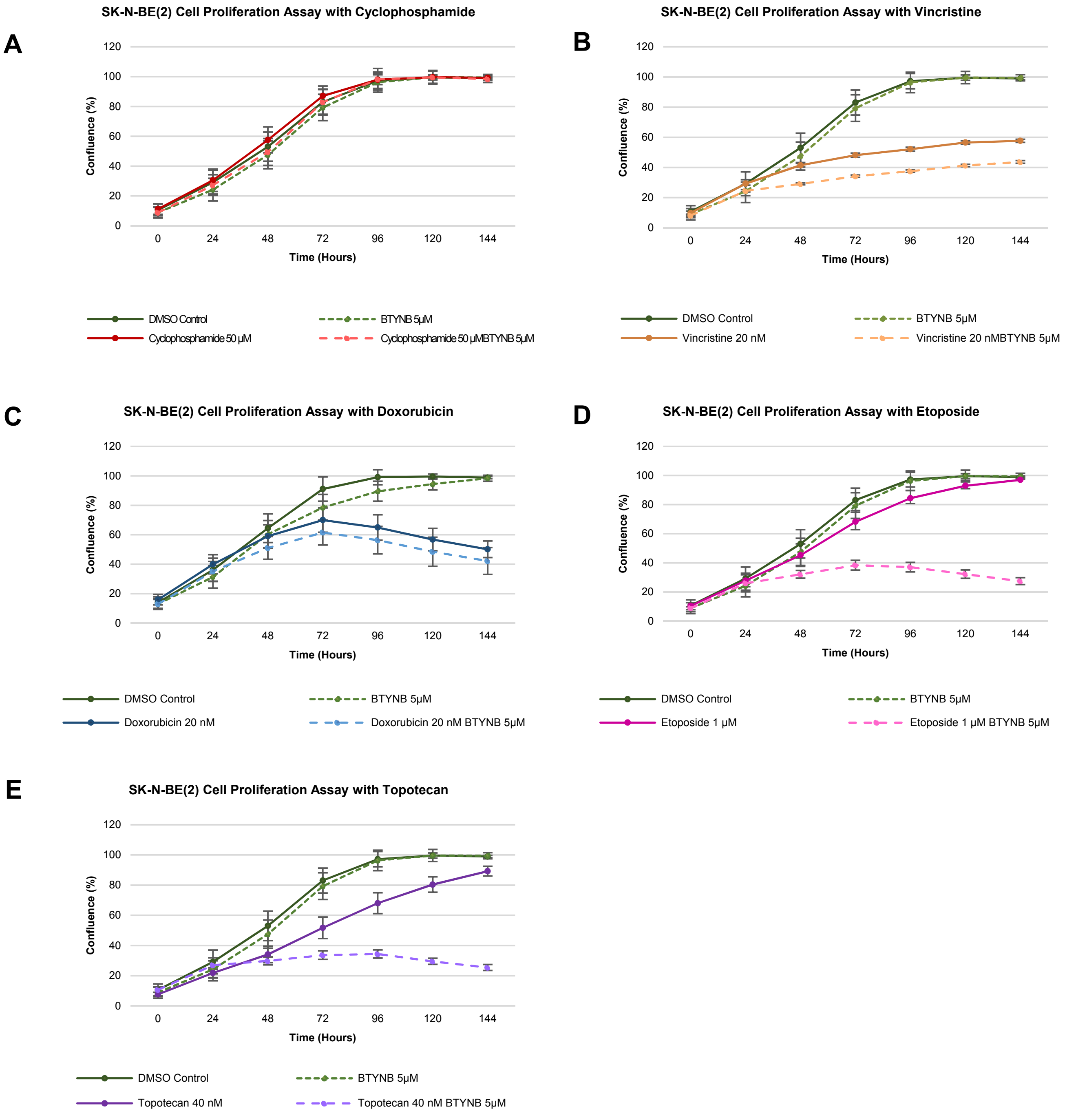

Supplementary Figure S6. IGF2BP1 inhibition sensitizes SK-N-BE(2) cells to chemotherapuetic agents

Comparison of the rate of growth of SK-N-BE(2) cells with BTYNB combined with multiple chemotherapeutic agents, including (A) Cyclophosphamide, (B) Vincristine, (C) Doxorubicin, (D) Etoposide, and (E) Topotecan. Each drug was tested separately and in combination as indicated and cell confluence was compared to the DMSO control over an interval of 6 days, n=3.

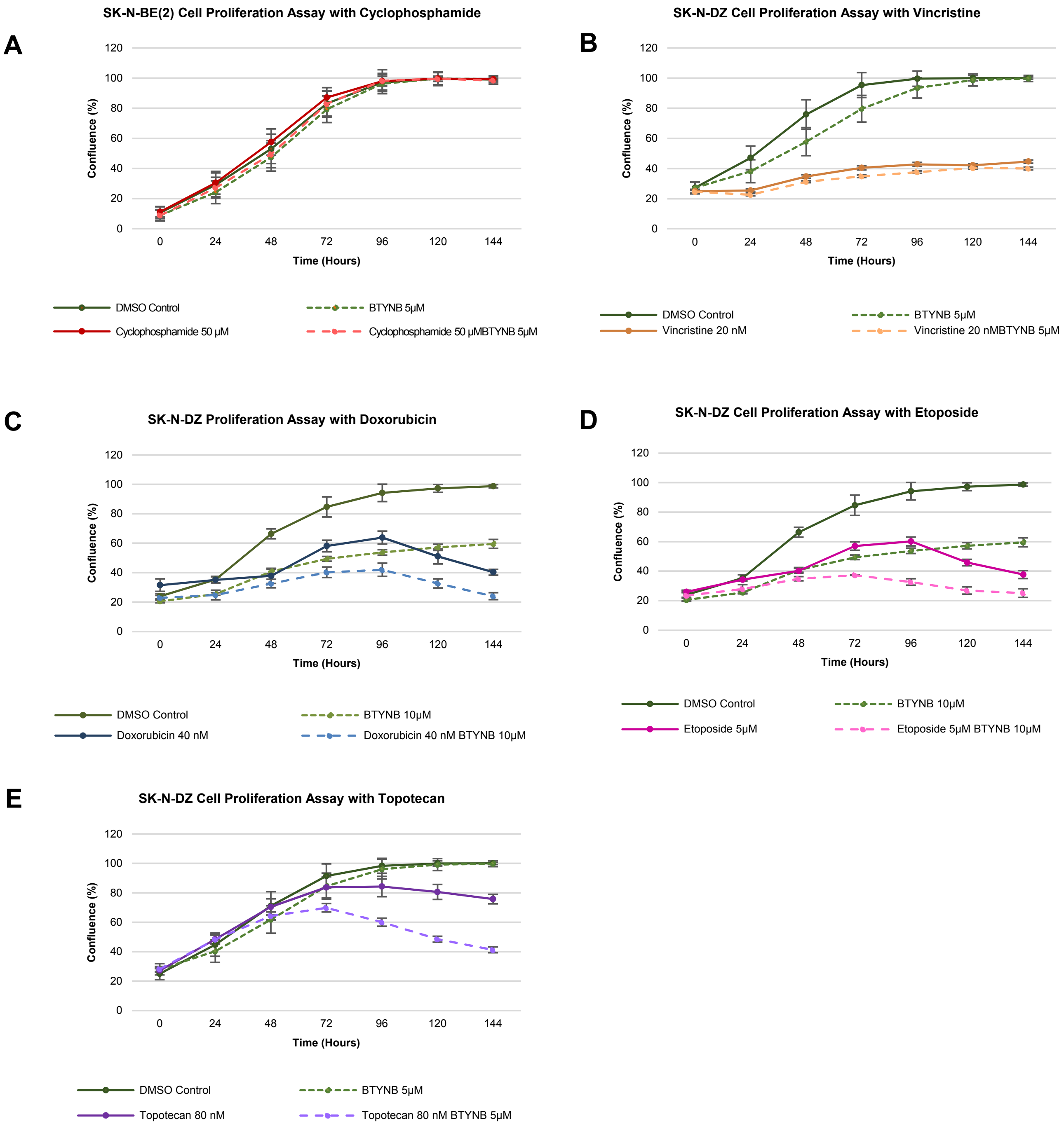

Supplementary Figure S7. IGF2BP1 inhibition sensitizes SK-N-DZ cells to chemotherapuetic agents

Comparison of the rate of growth of SK-N-DZ cells with BTYNB combined with multiple chemotherapeutic agents, including (A) Cyclophosphamide, (B) Vincristine, (C) Doxorubicin, (D) Etoposide, and (E) Topotecan. Each drug was tested separately and in combination as indicated and cell confluence was compared to the DMSO control over an interval of 6 days, n=3.
